# A prospective comparative study on bladder volume measurement with portable ultrasound scanner and CT simulator in pelvic tumor radiotherapy

**DOI:** 10.1007/s13246-023-01344-2

**Published:** 2023-11-29

**Authors:** Fei Bai, Qiuxia Hu, Xiaowei Yao, Ming Cheng, Lina Zhao, Linlin Xu

**Affiliations:** Department of Radiation Oncology, Xijing Hospital, Fourth Military Medical University, 127 West Changle Road, Xi’an, Shaanxi China

**Keywords:** Portable bladder scanner, Bladder volume, Pelvic neoplasm, Radiation therapy

## Abstract

Objective: The consistency of bladder volume is very important in pelvic tumor radiotherapy, and portable bladder scanner is a promising device to measure bladder volume. The purpose of this study was to investigate whether the bladder volume of patients with pelvic tumor treated with radiotherapy can be accurately measured using the Meike Palm Bladder Scanner PBSV3.2 manufactured in China and the accuracy of its measurement under different influencing factors. Methods: A total of 165 patients with pelvic tumor undergoing radiotherapy were prospectively collected. The bladder volume was measured with PBSV3.2 before simulated localization. CT simulated localization was performed when the bladder volume was 200-400ml. The bladder volume was measured with PBSV3.2 immediately after localization and recorded. The bladder volume was then delineated on CT simulation images and recorded. To compare the consistency of CT simulation bladder volume and bladder volume measured by PBSV3.2. To investigate the accuracy of PBSV3.2 in different sex, age, treatment purpose, and bladder volume. Results: There was a significant positive correlation with bladder volume on CT and PBSV3.2 (*r* = 0.874; *p* < 0.001). The mean difference between CT measured values and PBSV3.2 was (-0.14 ± 50.17) ml. The results of the different variables showed that the overall mean of PBSV3.2 and CT measurements were statistically different in the age ≥ 65 years, bladder volumes > 400ml and ≤ 400ml groups (*p* = 0.028, 0.002, 0.001). There was no statistical significance between the remaining variables. The volume difference between PBSV3.2 measurement and CT was 12.87ml in male patients, which was larger than that in female patients 3.27ml. Pearson correlation analysis showed that the correlation coefficient was 0.473 for bladder volume greater than 400ml and 0.868 for bladder volume less than 400ml; the correlation coefficient of the other variables ranged from 0.802 to 0.893. Conclusion: This is the first large-sample study to evaluate the accuracy of PBSV3.2 in a pelvic tumor radiotherapy population using the convenient bladder scanner PBSV3.2 made in China. PBSV3.2 provides an acceptable indicator for monitoring bladder volume in patients with pelvic radiotherapy. It is recommended to monitor bladder volume with PBSV3.2 when the planned bladder volume is 200-400ml. For male and patients ≥ 65 years old, at least two repeat measurements are required when using a bladder scanner and the volume should be corrected by using a modified feature to improve bladder volume consistency.

## Introduction

Radiotherapy (RT) plays a vital role in treating pelvic tumors. Technological advancements have made intensity-modulated radiation therapy (IMRT) the main external beam radiation technique for pelvic tumors [1, 2]. With its precise-targeted dose distribution and steep dose gradient between normal tissue and tumor, IMRT has improved the effectiveness of RT. For safeguarding the surrounding healthy tissue, it is essential to delineate the target volume and organs at risk accurately.

Bladder volume (BV) is a significant factor in determining the target volume for cervical/cervical cancer [3, 4], prostate cancer [5, 6], and rectal cancer [7, 8]. Variations in BV among treatment fractions have been observed; Ahmad et al. [4] found an average reduction of 71% in bladder capacity among patients with cervical cancer. Similarly, Chang et al. [7] demonstrated a 59% reduction in bladder capacity among patients with rectal cancer. These changes in BV lead to shifts in the target location [9, 10], resulting in the inaccurate delivery of the prescribed radiation dose and increasing the risk of small bowel and bladder complications [9]. Consistency in BV is essential to minimize the radiation therapy effects.

Patients are conventionally instructed to drink a fixed amount of water (400 to 800 mL) before each treatment session and wait wait the same time or until they are urged to urinate [4, 11]; this helps ensure a reproducible BV. However, despite these conventional methods, BV has a considerable variation [12, 13]. The clinical gold standard of therapy frequently includes comfortable bladder filling. This approach ensures a balance between bladder emptiness (maximizing patient comfort) and bladder fullness (pushing the small bowel out of the high-dose region) [14]. Nevertheless, the variability in bladder filling remains an ongoing challenge in many RT departments [4, 7, 14–17].

Therefore, a more objective approach to maintaining consistent BV is required to reduce the impact of bladder-volume changes on pelvic tumor radiation therapy. Daily Megavoltage Computed Tomography (MVCT) [5] and Cone Beam Computed Tomography (CBCT) [6] scans allow visualization of radiation targets and organs before each treatment. However, this method will only find that the filling material is insufficient when the patient is already on the treatment table. If the scan reveals inadequate bladder filling, patients must drink more water or wait until the bladder fills appropriately. Conversely, if the bladder is overfilled, patients are asked to empty it and drink water or, in some cases, to drain excess urine (at the patient’s request). Detecting suboptimal filling at this stage would impede the clinical workflow and cause treatment delays. However, this procedure is time-consuming, and repeated scans lead to increased radiation exposure and patient stress.

A portable bladder capacity tester, offers a viable solution to this problem. Having it as a surrogate would be ideal for streamlining the clinical workflow without incurring imaging doses or hindering the clinical workflow for patients with inadequate filling. While many investigational sites have studied the accuracy and clinical value of bladder scanners manufactured in the USA, no RT facilities have validated the accuracy of bladder scanners from China using large sample sizes. At our Organisation, we purchased Meike Palm Bladder Scanner PBSV3.2 *(Sichuan, China, Registration Certificate No.: Sichuan, CFDA 202,060,039)*. We aimed to improve the repeatability of BV when using this bladder scanner for RT planning and treatment of pelvic tumors. The goal was to minimize unnecessary repeated CBCT scans, reduce the dose to organs-at-risk (OARs), and alleviate the burden of bladder-filling patients. Our assessment focused on comparing the BV readings obtained from PBSV3.2 with those obtained from computed tomography (CT) scans and analyzing the differences. The results provide valuable insights to the RT department regarding the suitability of the Chinese-made bladder scanner PBSV3.2 for obtaining reproducible BV measurements in patients before RT for pelvic tumors, along with any discrepancies in BV identification and the scanning method used for different factors.

## Methods

### Patient population

Between 01 and 2022 and 07 Jul 2022, we prospectively selected 165 patients with pelvic tumors undergoing RT at our center. The inclusion criteria include Karnofsky Performance Score (KPS) ≥ 70, age ≥ 18 years, no contraindications to RT for pelvic tumors, and provision of written informed consent. The only exclusion criterion was the patient’s unwillingness to participate. The study received approval from the local Ethics Committee (KY20212191-F-1). Patient characteristics are presented in Table 1.

**Table 1:** General information of 165 patients

Demographics		N(%)
Age M(range)		55 (24–80)
Sex	Male	32 (19.4)
	Female	133 (80.6)
Place of residence	City	110 (66.7)
	Country	55 (33.3)
Disease	Cervical cancer	103(62.4)
	Endometrial carcinoma	9 (5.5)
	Rectal cancer	46 (27.9)
	Vulvar carcinoma	3 (1.8)
	Pelvic metastatic carcinoma	4 (2.4)
Surgery	No	95 (57.6)
	Yes	70 (42.4)

### Imaging equipment

**Fig. 1:**
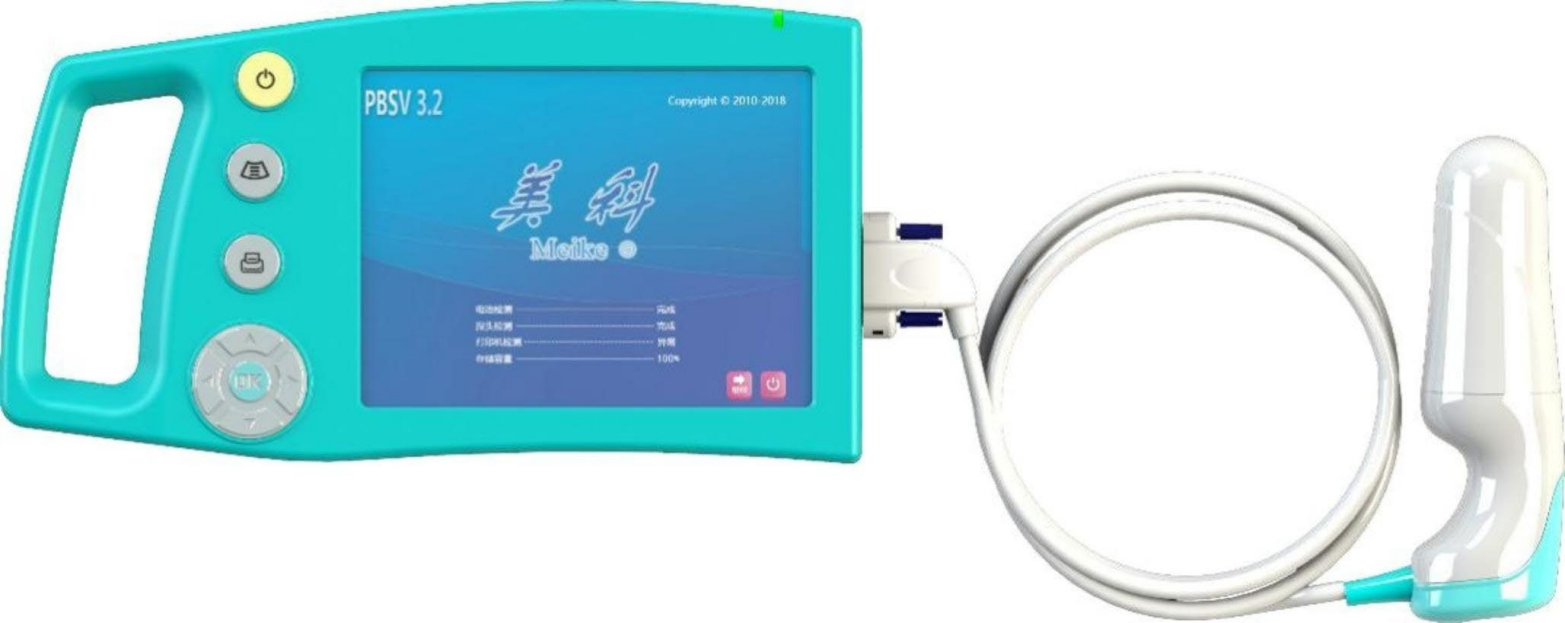
Meike® Palm Bladder Scanner PBSV3.2

The Meike® Palm Bladder Scanner (China) PBSV3.2 (Fig. 1) was used for BV measurements. The CT simulation was performed using Philips Brilliance TM Big Bore CT (Bore diameter 85 cm, 16 slices/360°Aperture size). Patients were positioned during CT simulation using the Belgian Orfit holder and Klarity Thermoplastic Body Film.

### CT simulation positioning process

Before the CT simulation positioning, all patients were provided with the institution’s positioning precautions, including instructions on preparation before positioning, conditions during positioning, and post-positioning care instructions. On the CT simulation positioning day, the patient was asked to empty their bladder and bowels. The patients were asked to drink 300–800 mL of water immediately (300–500 mL for patients who had breakfast in the morning; 500–800 mL for those who had not eaten), and the time point when they finished drinking water was noted.

The PBSV3.2 scan was performed when the patient experienced a subjective urge to urinate. When the scan results were > 100 mL, a fixed mold was made (approximately 20 min), and subsequently, the PBSV3.2 scan was repeated. The CT simulation positioned immediately when the scan results showed a BV 200–400 mL. If the scan results were < 200 mL, the wait was prolonged until the bladder was filled (immediately located if the patient could not do so); Above 400ml, some urine is excreted. If the patient subjectively desired to urinate and the measurement was < 100 mL, the patient was asked to wait; 400ml or more, the mold was made and CT simulation positioning after partial urine was discharged. The planned CT scan was performed in the treatment orientation, using a body plate and thermoplastic Body Film. A scan thickness of 5 mm was used, and the patient was scanned after receiving intravenous contrast. The scans cover the area from the upper pole of both kidneys to 5 cm below the ischial tubercle. Immediately after the scan, the PBSV3.2 measured and recorded the BV at that time, along with the time between drinking and the end of the scan. The images from the CT scan were transferred to the planning system, which automatically delineated the outer wall of the bladder. After review by the attending physician, BV was recorded; this value was considered to represent the actual volume of the bladder.

### Use of PBSV3.2 bladder scanner

**Fig. 2:**
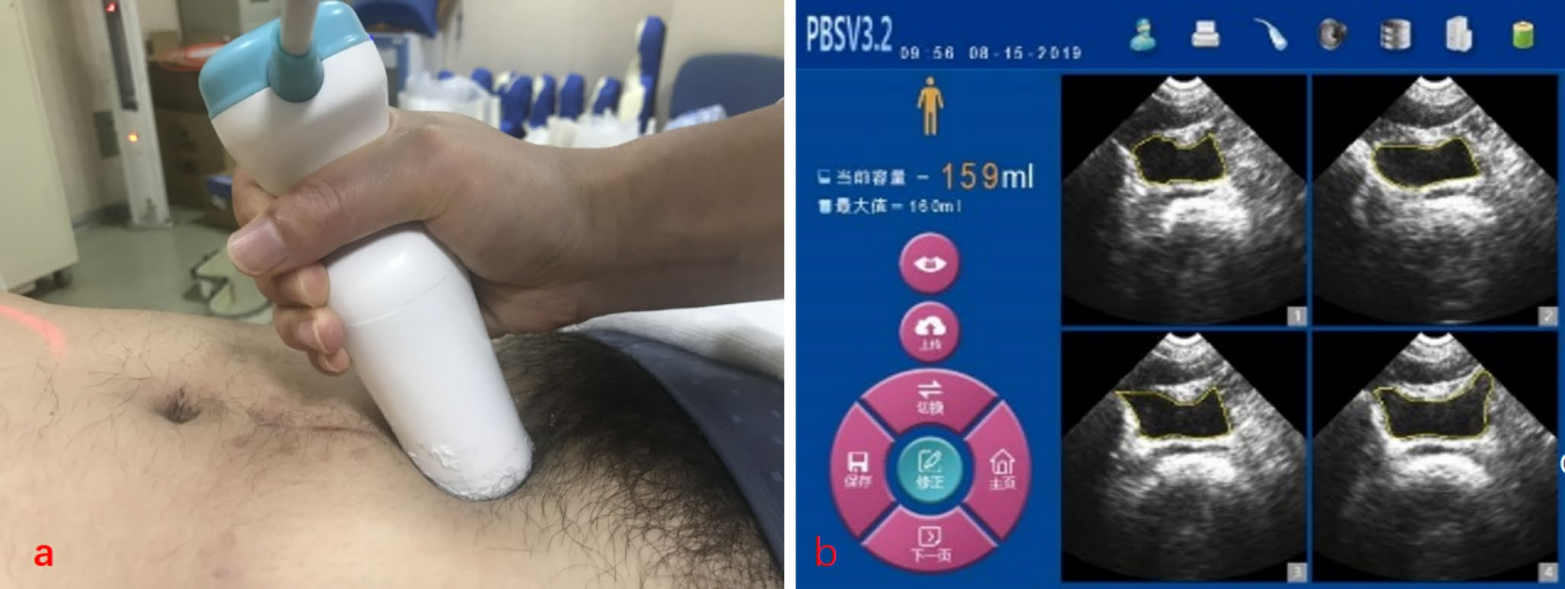
PBSV3.2 scanning screen. (**a**) The PBSV3.2 positioned for acquisition of bladder images. (**b**) Twelve bladder images in multiple planes that can be viewed after the scan data are loaded into the BladderScanÒ software

A CT simulation radiotherapist measured the BV using PBSV3.2. Select the appropriate scanning mode for patients of different genders(male mode or female mode)。The patient was laid supine during the examination, and an ultrasound coupling agent was applied approximately 3 cm above the pubic symphysis. With the CT simulation radiotherapist on the patient’s right side, the probe was aligned with the estimated bladder position, and the scan button was pressed to initiate the pre-scanning (Fig. 2a). Move the probe to find the largest area of the bladder fluid dark area, while making the bladder fluid dark area in the center area of the sector to determine the best scanning head position. After completing the measurement, it is recommended to review the dichroic image screen with the scan results to verify if the bladder outline coincides with the edge of the liquid dark area in the grayscale image (Fig. 2b). If the deviation is significant, manual correction is made, or the scan key could be pressed to rescan the measurement.

### Statistical analysis

The correlation between the PBSV3.2 and corresponding CT measurements for individual patients was assessed using Pearson correlation. Bland—Altman analysis [18] described the agreement between the two methods. The correlation coefficient quantifies the strength of the relationship between two variables and does not directly measure their agreement level. The Bland—Altman plots illustrate the difference in measurements against the mean and show the limits of agreement, providing a more appropriate measure of the clinical significance of differences between the measurement methods. BV measured by PBSV3.2 was compared to the BV from planning CT using a paired t-test. A two-sample T-test was used to analyze the differences between different variables. BV was presented as mean and standard deviation (SD). Statistical analyses were performed using IBM® SPSS® statistical software v.25.0.

## Results

### Comparison of bladder scanner measurements with CT measurements

**Fig. 3:**
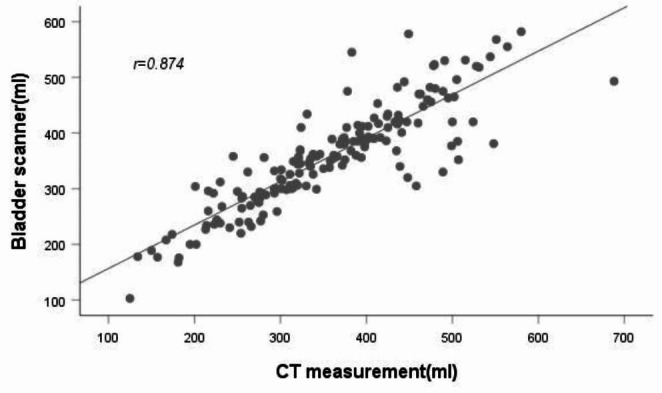
Scatter plot of the results of the two measurements

The mean BV measured by the PBSV3.2 was 358.28 ± 92.07 mL. The mean volume measured by CT was 358.14 ± 103.02 mL. There was no significant difference in the overall mean of estimated BV between the two groups (*t* = 0.036, difference and 95% CI: 0.139 (-7.57–7.85), *P* = 0.972). Pearson correlation analysis revealed a high correlation coefficient (*r* = 0.874, *P* < 0.001) between the two methods (Fig. 3).

### Difference comparison

**Fig. 4:**
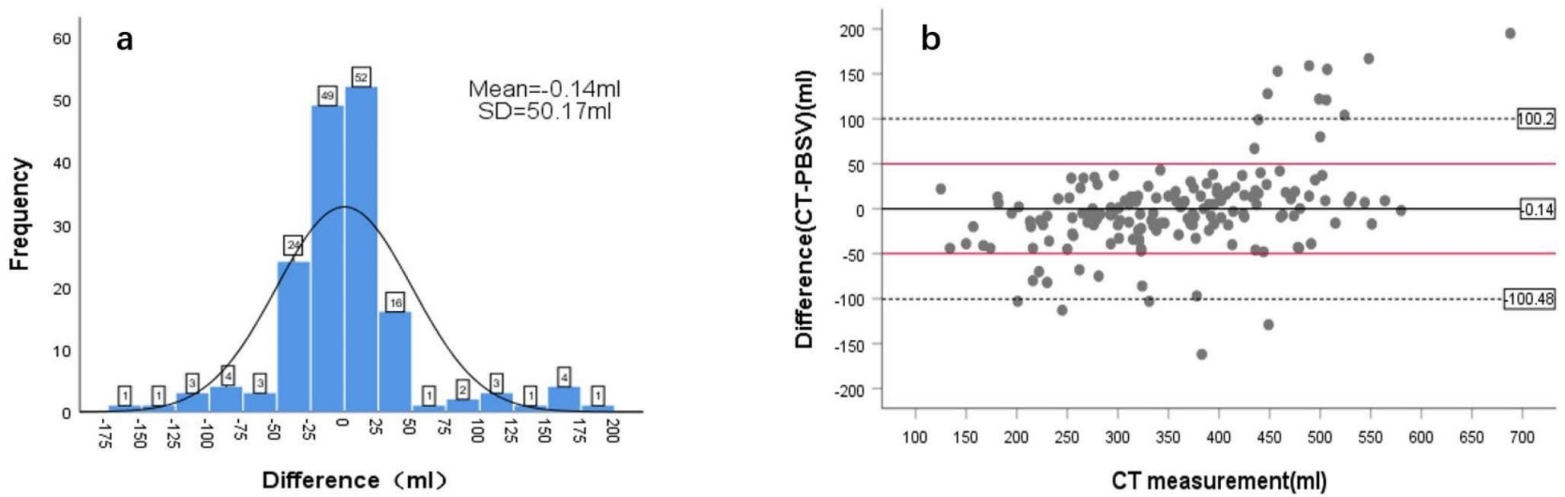
(**a**) Distribution plot of difference between CT and PBSV3.2 measurements; (**b**) According to the CT measured values and (CT-PBSV3.2) difference scatter diagram; draw a horizontal line at the mean difference; draw a horizontal dashed line at the mean difference ± 2 times the standard deviation of the difference; draw a horizontal red line at ± 50ml

**Fig. 5:**
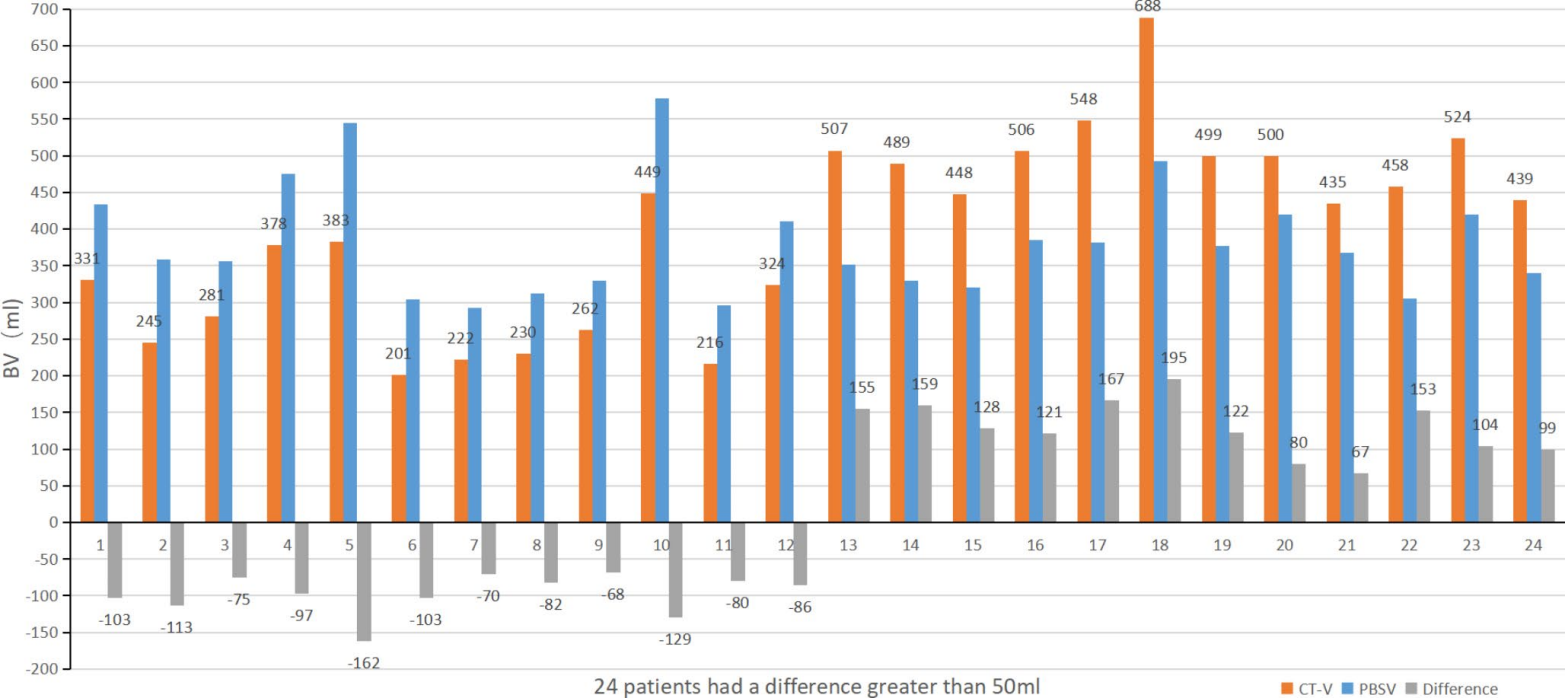
Bar chart of 24 patients’ BV with a difference greater than 50ml. Abbreviations: PBSV, Meike® Palm Bladder Scanner PBSV3.2; CT-V, CT Value

The difference between the CT-measured values and PBSV3.2 measurements was − 0.14 ± 50.17 mL, ranging from − 162 to 195 mL (Fig. 4a). Most differences (85.45% [141/165]) were ≤ 50 mL, while 14.54% (24/165) were > 50 mL. Among the differences, 12 measurements (ranging from 67 to 195 mL) of PBSV3.2 were less than those measured by CT, and 12 measurements (ranging from − 68 to 162 mL) were greater than those by CT. The PBSV3.2 measurements underestimated the actual BV as 435–688 mL in some cases and overestimated it as 201–449 mL in others (Fig. 5).

Figure 4b is a Bland-Altman scatter plot between CT measurements of BV and the difference between CT and PBSV3.2 measurements, representing the relationship between measurement error and actual value. The mean, mean ± 2SD, and mean ± 1SD are also marked in the plot.

### Accuracy of bladder scanner between genders

**Fig. 6:**
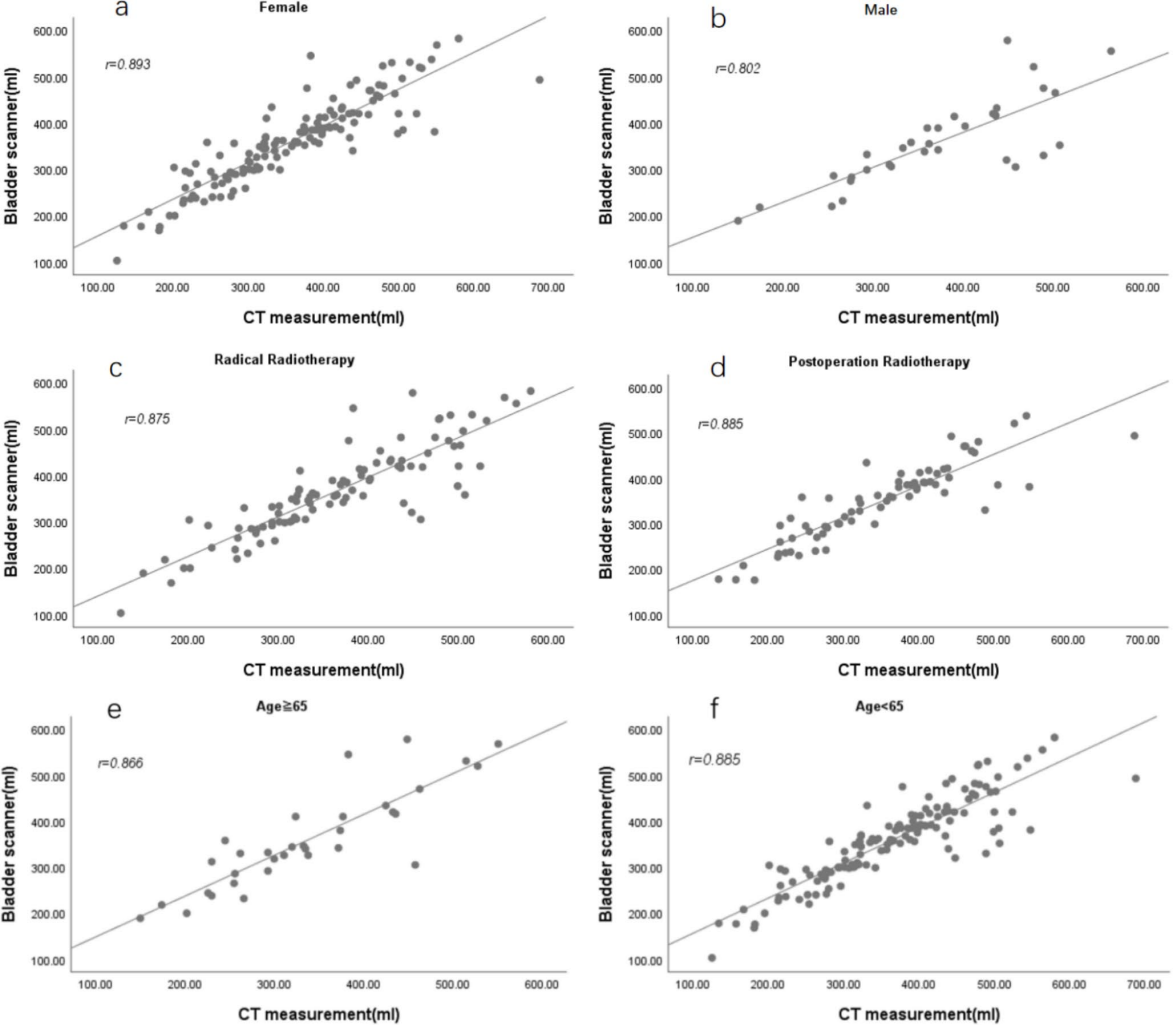
Pearson correlation plots of PBSV3.2 and CT measurements for different variables

Among 32 male patients, the mean BV measured by PBSV3.2 was 357.59 ± 93.96 mL, and that measured by CT was 370.47 ± 99.94 mL. There was no significant difference in the overall mean between the two groups (difference 12.87, 95% CI: -34.98–9.23, *P* = 0.244). Among 133 female patients, there was also no significant difference in the overall mean between the two groups (Table 2). Pearson correlation analysis revealed a high correlation (Fig. 6a and b).

**Table 2:** Comparison of measurement methods between genders (unit: ml)

Group	N	PBSV	CT-V	Difference and 95%CI	*P*-value
Male	32	357.59 ± 93.96	370.47 ± 99.94	12.87(-34.98-9.23)	0.244
Female	133	358.45 ± 91.97	355.18 ± 103.90	3.27(-4.76-11.30)	0.422
Difference and 95%CI		0.86 (-35.05-36.76)	15.29 (-55.40-24.82)		
P-value		0.962	0.453		

Abbreviations: PBSV, Meike® Palm Bladder Scanner PBSV3.2; CT-V, CT Value

### Accuracy of measurements between patients treated with radical radiotherapy (RR) and postoperative radiotherapy (PR)

Table 3 shows the difference between PBSV3.2 and CT measurements in patients undergoing RR and PR. The two groups showed no significant differences in the overall and inter-group mean for PBSV3.2 and CT measurements. Pearson correlation analysis demonstrated a high correlation between the two measurements (Fig. 6c and d).

**Table 3:** Comparison of two measurement methods for RR and PR patients (unit: ml)

Group	N	PBSV	CT-V	Difference and 95%CI	*P-value*
RR	95	365.91 ± 97.40	364.43 ± 99.81	1.48(-8.58-11,55)	0.770
PR	70	348.01 ± 83.90	349.61 ± 107.37	1.60(-13.82-10.62)	0.795
Difference and 95%CI		17.90 (-10.69-46.49)	14.82 (-17.24-46.88)		
*P*-value		0.218	0.363		

Abbreviations: RR, radical radiotherapy; PR, postoperative radiotherapy

### Comparison of the difference between the two measurement methods in different age groups

In those aged ≥ 65 years, the mean BV measured by PBSV3.2 was 358.33 ± 106.15 mL, and that by CT was 336.57 ± 103.66 mL. There was a significant difference in the overall mean between the two groups (difference 21.76, 95% CI: 2.49–41.03, *P* = 0.028). In those aged < 65 years, there was no significant difference in the overall mean between the two groups (Table 4). Pearson correlation analysis revealed a high correlation between the two methods (Fig. 6e and f).

**Table 4:** Comparison of two measurement methods in ml between patients aged ≥ 65 years and < 65 years (unit: ml)

Group	N	PBSV	CT-V	Difference and 95%CI	*P*-value
Age ≥ 65	33	358.33 ± 106.15	336.57 ± 103.66	21.76(2.49–41.03)	0.028*
Age < 65	132	358.27 ± 88.66	363.54 ± 102.55	5.26(-13.49-2.96)	0.208
Difference and 95%CI		0.061 (-35.43-35.55)	-26.962 (-66.46-12.53)		
*P*-value		0.997	0.180		

* P < 0.05

### Comparison of BV measurements above and below 400 mL: a comparative analysis of PBSV3.2 and CT

Among the patients had with BV > 400 mL (n = 57), the mean value measured by PBSV3.2 was 443.26 ± 65.37 mL, while that measured by CT was 470.33 ± 53.95 mL. The difference between the two groups was significant (difference − 27.07, 95% CI -43.53 to -10.61, *P* = 0.002) (Table 5). Correlation analysis indicated a Pearson correlation coefficient of 0.473 for BVs > 400 mL and 0.868 for those < 400 mL.

**Table 5:** Comparison of BV measurements above and below 400 mL (unit: ml)

Group	N	PBSV	CT-V	Difference and 95%CI	*P*-value
≥ 400	57	443.26 ± 65.37	470.33 ± 53.95	-27.07(-43.53/-10.61)	0.002*
≤ 400	108	313.43 ± 69.86	298.93 ± 67.09	14.50(7.78/21.22)	0.001*

* P < 0.05

### Number of PBSV measurements

The proportion of bladder volume measurements using PBSV3.2 to meet the requirements of CT simulation positioning was 7.27% (12/165) for one measurement, 43.64% (72/165) for two measurements, and 49.09% (81/165) for three or more measurements. It can be seen that 92.73% of patients needed PBSV3.2 measurement twice or more to achieve the bladder volume for CT simulation localisation.

## Discussion

This study compared BV measurements in 165 patients undergoing RT for pelvic tumors using PBSV3.2, manufactured in China, and CT scans. To our knowledge, this is the first large-scale study in RT to investigate the accuracy of a Chinese-made bladder scanner. The results demonstrated that PBSV3.2 could effectively measure BV in pelvic tumor RT, ensuring consistency during CT simulation localization and before each RT session. There was a strong correlation between BV measured by PBSV3.2 and CT scans (*r* = 0.874). Yoon et al. [19] and Chang et al. [7] conducted separate studies involving 20 patients with rectal cancer receiving RT, demonstrating a strong correlation between BV measured by BioCon-700 (Mcube Technology, Seoul, Korea) and CT (*r* = 0.87, 0.93). Claxton et al. [20] and Smith et al. [6] measured BV in 20 patients with cervical/endometrial cancer and 19 patients with prostate cancer treated with RT, respectively, using BioCon-700 and CT, and obtained similar strong correlations. Stam MR et al. [14] and O’Doherty et al. [21] enrolled 26 and 41 patients with prostate cancer, respectively, and showed a strong correlation between BV measured by BladderScan BVI3000(Verathon Medical Europe, Washington State, USA) and CT (*r* = 0.95, *r* = 0.88). Kuo et al. [5] and Mullaney et al. [22] employed BVI6100 and CT to measure BV in 11 and 190 patients with prostate cancer receiving RT, respectively; correlation coefficients of 0.87 and 0.83 were obtained. Ahmad et al. [4] and Hynds et al. [16] used BVI6400 and CT to measure BV in 24 patients with cervical cancer and 30 patients with prostate cancer, respectively, and observed correlation coefficients of 0.97 and 0.91. Luo et al. [23] and Cramp et al. [24] employed BVI9400 to measure BVs in 13 patients with cervical carcinoma undergoing RT and 34 patients with prostate carcinoma; they obtained correlation coefficients of 0.95 and 0.80, respectively. According to the above studies, the correlation between bladder scanner measurements and those of CT decreases as the number of cases increases. Our study benefits from a substantial patient sample size, which provides a more accurate representation of the accuracy of the bladder scanner.

In this study, the mean BV measured by PBSV3.2 was 358.28 ± 92.07 mL, and that delineated by CT was 358.14 ± 103.02 mL, resulting in a mean difference of -0.14 ± 50.17 mL (*P* = 0.972). Moreover, within a minute, the PBSV3.2 scans were performed immediately after the completion of the CT scan, which minimizes any volume differences due to time discrepancies. BV delineated by CT at our institution includes the entire bladder, bladder wall, and urine, which might result in a slightly higher volume compared to the actual BV. The CT-based BV, specifically based on the inner bladder wall contour, could provide a closer approximation to the urine volume. However, determining the urine volume accurately based on the inner wall delineation is challenging due to the poor visibility of the inner wall [4, 22]. Moreover, a high Pearson correlation coefficient (*r* = 0.97) has been observed in cases with and without bladder wall inclusion [4]. The consensus among radiation therapists is to use outside wall delineation, considering it the current standard. The difference between the bladder scanner and CT measurements of BV reported in other studies is consistent with our findings. The mean difference elicited by Claxton et al. [20] (CT-US) was − 10 ± 49.92 mL, -16 ± 67 mL by Ahmad et al. [4], 6.5 ± 48.8 mL by Luo et al. [23], -9.7 ± 64.6 mL by O’Shea et al. [25] (n = 50), and 9.0 ± 47 mL by Hynds et al. [16].

The SD of the difference between CT and BVI measurements could be used as the standard to evaluate the accuracy of the scanner [22]. Previous studies have reported SD values of 47 mL [16] and 64.6 mL [25] in patients with prostate cancer and 48.8 mL [23], 49.92 mL [20], and 67 mL [4] in patients with cervical cancer. In our study, the SD of the difference between CT and PBSV measurements was 50 mL (1SD), which could be considered as a measure of the accuracy of PBSV3.2 (Fig. 4b). Considering the bladder changes in the uterine/cervical position, the value of 1SD is preliminarily used as the maximum allowable relative deviation of BV for fractionated RT. The clinical interpretation of these accuracy levels indicates that the PBSV exhibits some inaccuracy compared to the described BVs. This imprecision was particularly evident in 24 patients (Fig. 5). Among them, the CT volume (201–449 mL) was smaller than the PBSV volume (292–578 mL) for 12 patients, with a difference of -68 to -162 mL, while for the remaining 12 patients, the CT volume (435–688 mL) was larger than the PBSV3.2 volume (305–493 mL), with a difference of 67 to 195 mL. Analysis of these patients revealed that PBSV3.2 measurements were larger than CT values, primarily due to the gut surrounding the periphery of the bladder, making PBSV3.2 indistinguishable. Additionally, the scans did not correct these measurements, which could be attributed to the scanner’s skills. Among the instances where PBSV3.2 measurements were lower than CT values, 11 cases were attributed to the bladder’s overcapacity and irregular shape (anteroposterior, left and right diameter, or superoposterior diameter), hindering the entire bladder scanning. One case was due to a low coupling agent or operator technique issue. Our data (Table 5) suggested that PBSV3.2 tends to underestimate BV (-27.07 mL) when the volume is > 400 mL in patients undergoing pelvic irradiation and overestimate it (14.5 mL) when BV is < 400 mL. Considering the repeated action of bladder filling during treatment and the patient’s comfort, we recommend controlling the planned BV at 200–400 mL when using a bladder scanner. Analysis showed a strong correlation between PBSV and CT measurements for BV between 200 and 400ml (r = 0.86), with a SD of 36ml. It can be seen that the PBSV measurement will be closer to the true value with BV in its range. Further analysis found that the PBSV accuracy level was 60.3 ml when the planned BV was outside 200-400ml, with a correlation coefficient of 0.66. It can be seen that the accuracy of the PBSV measurement decreases when the planned BV is within its range. Although our PBSV3.2 operator initially received only a brief tutorial from the manufacturing engineer, our results showed a high correlation of PBSV3.2 measurements even when operated by non-professional ultrasound technicians. With practice and experience, the consistency of PBSV3.2 measurements could improve.

In pelvic RT, the dose and volume of OARs, such as the small intestine, limit the ability to increase the local tumor dose. Studies show that bladder filling state is negatively correlated with the volume and dose of small intestine irradiated; It is associated with acute intestinal adverse reactions [26, 27]. Therefore, optimal bladder filling is crucial in pelvic tumor RT. However, a large number of studies [4, 7, 16, 29] found that bladder volume decreased significantly with the advancement of treatment (33%, 38%, 71%, 59%). It can be seen that the patient cannot repeat the BV at the time of planning during treatment, and the difference is relatively large. This leads to underdose in the target area and increase of toxic side effects. All of these studies performed detailed bladder filling training, either orally or in writing. Therefore, the bladder volume is not easy to be too large in CT simulation. Bózsa et al. [29] suggested that a planned BV between 200 and 400 mL is acceptable. Smith[6] believes that the target BV of at least 200 ml should be achieved in the planning stage, and the BV measured by CBCT scan and ultrasound should be at least 50% of the planned volume before each treatment. Eminowicz et al. [30] recommends a planned BV of 150–300ml; A maximum of 50ml less or 150 ml more BV than planned is allowed during treatment. Therefore, our study suggests a planned bladder capacity of 200–400 mL similar to the results of existing studies of bladder volume consistency during treatment. This will support from another point of view that when the bladder capacity of patients with pelvic tumor radiotherapy is 200–400 ml, the inter-fraction repeatability is better.

Several studies [31–33] have highlighted the importance of bladder filling in cervical mobility. Bladder filling has a greater effect on the uterus than on the cervix [30, 32]. With bladder filling, the uterine motion range is 5–40 mm in the head-foot direction and 0–65 mm in the anteroposterior direction [34]. BV influences the displacement, deformation, or rotation of adjacent organs, ultimately affecting the accuracy of target localization and the margins between clinical target volume (CTV) and planned target volume (PTV) [35, 36]. Similarly, changes in bladder volume during radiotherapy for rectal cancer have an impact on target margin and intestinal dose [15, 37]. Other studies have shown that the change of bladder volume during treatment leads to an increase in treatment setup error, and a relatively consistent bladder volume can improve treatment accuracy [38]. Therefore, maintaining a consistent BV is necessary for fractionated RT. Through the analysis of the number of PBSV measurements for each patient, it was found that if there is no PBSV, according to the traditional method, in order to meet the requirements of bladder capacity during planning, the patient will have to repeat the CT simulation scan for many times, resulting in the increase of radiation dose, positioning time and other adverse factors. The same is true in fractionated radiotherapy. It can be seen that PBSV would be an ideal surrogate tool for maintaining consistent bladder volume. The PBSV3.2, with its advantages of high accuracy, small size, ease of operation, zero radiation, non-invasiveness, and rapid process, provides a convenient method for identifying BV discrepancies before treatment planning and daily RT sessions. Most importantly, The device improves workflow efficiency and treatment accuracy in the RT department.

At our site, RT for pelvic tumors requires moderate bladder filling. Patients are informed through written notifications and oral reminders on the CT simulation positioning day. After emptying their bladder and bowels one hour before CT simulation positioning and each RT session, they are instructed to drink a specified amount of water (350 or 800 mL). This protocol aligns with practices in other institutions. However, BV still exhibits significant variability during RT due to factors such as diet, water intake, and patient age [8]. To evaluate these differences, we analyzed the discrepancies in BV measured by PBSV3.2 and CT based on gender, treatment objectives (PR and RR), and age groups. No significant differences were found between or within groups for genders and treatment objectives. However, there was a significant difference in BV between PBSV3.2 and CT for patients aged ≥ 65 years (difference 21.76, 95% CI: 2.49–41.03, *P* = 0.028), whereas no significant difference was observed in patients aged < 65 years. Correlation analysis indicated that the correlation was lowest when the planned BV was > 400 mL (correlation coefficient of 0.473), followed by 0.802 in men and 0.893 in women. The lower correlation in cases with a planned BV of > 400 mL was primarily due to an oversized bladder. The lower correlation in men could be attributed to subcutaneous muscle and deeper bladder location. For patients aged ≥ 65 years, the presence of intestinal gas might have led to overestimated PBSV3.2 measurements. Therefore, PBSV3.2 scans should be performed with slightly stronger pressure, repeated at least twice, and utilizing the correction function to achieve more consistent BVs. Furthermore, manufacturers of PBSV3.2 should continuously optimize the precision of the devices to accurately identify bladder boundaries and minimize the impact of intestinal gases, intestinal fluids, and uterine bodies.

There are some limitations to this study. Firstly, the bladder scanners were operated by three radiotherapist involved in the CT simulation process, each of whom had received < 10 min of training from the manufacturing engineers. The skill level of each radiotherapist could have influenced the accuracy of the scan results, some radiotherapist determine BV from only one scan, These potentially underestimating the accuracy of PBSV3.2. Whether using the average of two scans can improve the accuracy of scanning will be the focus of the next study. Secondly, the study did not account for the effects of diet, water intake, bowel preparation, and body mass index (BMI) on the accuracy of BV scanning. Although the research by Kuo et al. [5] indicated that BMI did not significantly affect the accuracy of Bladder Scan measurements, it would be valuable to investigate the impact of these factors on the accuracy of PBSV3.2 measurements in future studies. Finally, the analysis of individual cases for different variables was limited by a small sample size (*N* = 32), which may have introduced bias into the results. Therefore, further validation through larger studies is anticipated.

## Conclusions

The comparison between BV delineated by the PBSV3.2 and CT demonstrated a strong correlation between the two measurement methods. In the context of RT for pelvic tumors, the PBSV3.2 tends to underestimate BV when it is > 400ml, while it tends to overestimate the volume when it falls below 400 mL. For male patients ≥ 65 years of age, we recommend repeating the PBSV3.2 scan multiple times and utilizing the correction function. When monitoring BV with the PBSV3.2, a planned BV in the 200–400 mL range is preferred. Overall, PBSV3.2 serves as a satisfactory tool for monitoring bladder filling in patients undergoing pelvic tumor RT and simplify clinical workflow.
